# Risk of Fetal Death after Treatment with Antipsychotic Medications during Pregnancy

**DOI:** 10.1371/journal.pone.0132280

**Published:** 2015-07-10

**Authors:** Merete Juul Sørensen, Maiken Ina Siegismund Kjaersgaard, Henrik Søndergaard Pedersen, Mogens Vestergaard, Jacob Christensen, Jørn Olsen, Erik Parner, Lars Henning Pedersen, Bodil Hammer Bech

**Affiliations:** 1 Regional Center for Child and Adolescent Psychiatry, Aarhus University Hospital, Aarhus, Denmark; 2 Section for Biostatistics, Department of Public Health, Aarhus University, Aarhus, Denmark; 3 Research Unit for General Practice, Department of Public Health, Aarhus University, Aarhus, Denmark; 4 Section for General Medical Practice, Department of Public Health, Aarhus University, Aarhus, Denmark; 5 Department of Neurology, Aarhus University Hospital, Aarhus, Denmark; 6 Department of Clinical Pharmacology, Aarhus University Hospital, Aarhus, Denmark; 7 Section for Epidemiology, Department of Public Health, Aarhus University, Aarhus, Denmark; 8 Institute of Clinical Medicine—Obstetrics and Gynaecology, Aarhus University, Aarhus, Denmark; University of Manitoba, CANADA

## Abstract

**Background:**

Antipsychotic medications are increasingly used during pregnancy. Nevertheless, fetal risks are still not fully studied. It is currently unclear whether the antipsychotic treatment might induce a higher risk of fetal death. We aimed to determine if use of antipsychotic medication during pregnancy is associated with an increased risk of spontaneous abortion or stillbirth.

**Methods:**

In a historical cohort study, we identified all clinically recognized pregnancies registered in the nationwide Danish registries from 1997 to 2008 (N = 1,005,319). Exposure was defined as any prescription of antipsychotic medications redeemed by the pregnant women during the exposure window, and recorded in the Danish National Prescription Register. Outcome was defined as any spontaneous abortion or stillbirth recorded in the Danish National Hospital Register and the Danish Medical Birth Register respectively.

**Results:**

Women exposed to antipsychotic medications during pregnancy had a 34% higher risk of spontaneous abortion (adjusted relative risk = 1.34; 95% confidence interval = 1.22; 1.46) compared to unexposed women, but a similar risk compared to women exposed prior to (but not during) pregnancy (adjusted relative risk = 1.04; 95% confidence interval = 0.93; 1.17). The risk of spontaneous abortion was not increased in exposed pregnancies when compared to unexposed pregnancies in the same women (adjusted hazard ratio = 1.11; 95% CI = 0.94; 1.31). A twofold higher risk of stillbirth was found in women exposed to antipsychotic medications compared with unexposed women (relative risk = 2.27; 95% confidence interval = 1.45; 3.55) and compared with women exposed only prior to pregnancy (relative risk = 2.06; 95% confidence interval = 1.01; 4.19).

**Conclusions:**

The increased risk of spontaneous abortion found in women treated with antipsychotic medications during pregnancy is most likely due to confounding factors. The risk of stillbirth was twofold higher in pregnancies exposed to antipsychotic medication during pregnancy. Treatment with antipsychotic medications during pregnancy requires careful consideration.

## Introduction

Women with severe psychiatric disorders, including schizophrenia, are at increased risk of pregnancy-related complications such as premature delivery, low birth weight, stillbirth [1–3] and induced abortion. [4] However, the underlying reasons are poorly understood.

Treatment of pregnant women with antipsychotic medication presents an important clinical challenge. [5–7] As antipsychotic medications may pass the placenta barrier, [8] fetotoxic effects are possible, but the potential impact is not well described. [9] Preterm birth, [10, 11] birth complications, congenital malformations [12–14] and impaired motor functioning in the newborn child [15, 16] have been reported, but information about spontaneous abortion (SA) and stillbirth is sparse. In a large cohort study, Habermann et al found no significantly increased risk of SA in 561 pregnancies exposed to second-generation antipsychotics (SGA) compared with 284 pregnancies exposed to first-generation antipsychotics (FGA) and 1122 pregnancies unexposed to medication with potential teratogenic effects. [14] Only five stillbirths occurred; two in the cohort exposed to FGA and three in the unexposed cohort. Two smaller studies (N = 215 and N = 151) found no increased risk of SA or stillbirth after prenatal exposure to atypical antipsychotic medications. [17, 18] However, both studies were subject to potential bias due to recruitment via self-referral to counselling services. Other papers reporting on risk of SA or stillbirth after prenatal exposure to antipsychotic medications are mainly based on surveillance studies; none of these include comparison groups [19–22] and most are based on only few pregnancies. [19–21]A large Danish cohort study found an increased risk of post-neonatal death and a marginally increased risk of congenital malformations in children born by women diagnosed with schizophrenia, but found no significantly increased risk of stillbirth or neonatal death. [23] However, this study did not include prenatal exposure to antipsychotic medications.

We conducted a large population-based retrospective cohort study to estimate associations between maternal use of antipsychotic medications during pregnancy and SA or stillbirth while adjusting for potential confounders including prenatal maternal psychiatric disorder. To further disentangle the potential effect of antipsychotic medications and possible confounding factors, we compared the outcome of exposed pregnancies with the outcome of pregnancies in which the mother redeemed a prescription to antipsychotic medications prior to, (but not during) the pregnancy. We further performed a study in women with multiple pregnancies discordant for antipsychotic medications exposure, comparing exposed with non-exposed pregnancies.

## Methods

### Study population

We identified all clinically recognized pregnancies in Denmark with an estimated conception date and an observed pregnancy outcome in the period from 1 February 1997 to 31 December 2008. Information was obtained from the nationwide Danish health registries, which include information about all pregnancies, except for very early miscarriages, which may be considered a late menstrual period. Information was linked through the Danish personal identification number, assigned to all citizens. We investigated all inpatient or outpatient contacts involving a diagnosis of spontaneous abortion before 22 weeks of gestation (in Denmark, a child born after 22 weeks of gestation is either stillborn or live born). We also included specific information on pregnancies ending in a molar pregnancy, ectopic pregnancy, induced abortion, stillbirth or live birth.

The pregnancy period was defined as the time from the estimated date of conception to the date of the outcome (induced or spontaneous abortion, stillbirth or live birth). Date of conception was estimated by subtracting gestational age at pregnancy outcome from date of outcome (induced or spontaneous abortion, stillbirth or live birth). For all abortions (spontaneous or induced), the gestational age was based on records in the Danish National Hospital Register. For live births and stillbirths the gestational age was based on records in the Medical Birth Register. Gestational age of less than 12 weeks was generally based on the last menstrual period, while age above 12 weeks was usually based on ultrasound scans. In case of missing data, gestational age was imputed based on the median of the available values.

We performed a hierarchical coding of the pregnancies to take repeated contacts into consideration. Any stillbirth or live birth resulted in recoding of former endpoints in the index pregnancy period. Pregnancies with multiple codes for abortion were coded as a SA if indicated by the pattern of codes, even for cases in which the index period also included codes for induced abortion (because a SA might occur in the time period between the initial contact for induced abortion and the planned surgical termination).

The primary outcome measures were 1) diagnosis of SA before 22 weeks of gestation and 2) diagnosis of stillbirth.

### Medication exposure

All antipsychotic medications are given by prescription in Denmark. The Danish National Prescription Register [24] holds information on all redeemed prescriptions administered as part of the Danish universal health-care system since 1 January 1996. However, the register does not contain information about medical treatment given only during inpatient hospital admission.

The medication exposure window was defined as the period from 30 days before the estimated conception date to one day prior to spontaneous abortion/stillbirth/birth. We included the 30 days prior to conception to investigate delayed medication effects and to account for prescriptions in use at the time of conception. We included the day prior to termination of the pregnancy to investigate any possible immediate effects of medication. In the SA analyses, the exposure window ended on the day before the gestational age limit for SA, i.e. 152 days. In sensitivity analyses we extended the exposure window to include the 6 months prior to conception. We defined exposed women as women who redeemed a prescription of antipsychotic medications (Anatomical Therapeutic Chemical (ATC) code: N05A) during the exposure window (3,164 pregnancies, 0.32%). We specifically assessed risks for a number of individual drugs (Chlorprothixene: N05AF03, Flupentixol: N05AF01, Perphenazine: N05AB03, Zuclopenthixol: N05AF05, Levomepromazine: N05AA02, Quetiapine: N05AH04, Olanzapine: N05AH03, Lithium: N05AN01, Risperidone: N05AX08, Aripiprazole: N05AX12, Ziprasidone: N05AE04, Prochlorperazine: N05AB04, Fluphenazine: N05AB02, Chlorpromazine: N05AA01). We defined unexposed women as pregnant women who did not redeem any prescription of antipsychotic medications during the exposure window. We estimated the average daily dose of antipsychotic medications during pregnancy as the total amount of redeemed antipsychotic medications divided by the number of days in the exposure window. The Defined Daily Dose (DDD) was defined as “the assumed average maintenance dose per day for a drug used for its main indication in adults” as also established by the World Health Organization (WHO) (http://www.whocc.no/ddd). Based on the DDD, the estimated daily antipsychotic medication dose was dichotomized into high (>50% of DDD) and low (≤ 50% of DDD).

### Pregnancy outcome

Abortions were identified in the Danish National Hospital Register, [25] which contains data on all inpatient and outpatient contacts in Denmark since 1995 coded according to the International Classification of Diseases, 10^th^ revision (ICD-10) [26] Abortions (ICD-10: O02.0-O06.9) were divided into spontaneous abortions (ICD-10: O02.0-O03.9), induced abortions (ICD-10: O04.0-O05.2, O05.5-O06.9) and induced abortions due to fetal disease (ICD-10: O05.3, O05.4). Molar or ectopic pregnancies (ICD-10: O00.0-O01.9) were excluded from the main analyses. Furthermore, failed induced abortions (ICD-10: O07) were disregarded as we assumed that a failed induced abortion would be followed by another abortion, a stillbirth or a live birth, which were all diagnosed separately. Live births and stillbirths were identified in the Danish Medical Birth Register, which holds information on all births in Denmark. [27] The definition of stillbirth changed in Denmark in 2004 from fetal death after 28 weeks of gestation to 22 weeks. Therefore, spontaneous abortions with gestational age (GA) of 22 weeks or more, were recoded as stillbirths in this study.

### Maternal psychiatric disorder and covariate information

The Danish Psychiatric Central Register [28] was used to identify mothers diagnosed with psychiatric disorder anytime before the end of pregnancy. The Danish Psychiatric Central Register includes information on treatment at psychiatric hospital-based units in Denmark since 1969 and outpatient contacts since 1995. Before 1994, psychiatric disorders were coded according to the ICD-8. From January 1994 and onwards they were coded according to the ICD-10. The register does not include diagnoses made by general practitioners or private psychiatrists.

History of severe mental disorder was defined as a diagnosis of bipolar disorder, including mania or schizophrenia (ICD-10: F30-F31 and F20; ICD-8: 296.1–296.8, 298.1 and 295), and history of misuse was defined as a diagnosis of alcohol or drug abuse (ICD-10: F10-F19; ICD-8: 291, 303, 304 and 294.3) at any time before the end of the index pregnancy.

Information on the following potential confounders was obtained from Statistics Denmark: maternal age at conception, cohabitation at time of conception, income at time of conception and education level at time of conception. From the Danish National Prescription Register, we obtained information on use of other medications.

### Analytic approach

Risk ratios (RR) for SA and stillbirth were estimated by binomial regression (i.e. we fitted a generalized linear model for our binary outcome and used a log-link) with robust variance estimation to allow for correlation between pregnancy outcomes of each woman. Pregnancies ending in induced abortion were excluded from the analyses (1,164 exposed pregnancies, 176,734 unexposed pregnancies). RRs for SA were adjusted (aRR) for maternal age (corresponding to the three tertiles), history of misuse (yes/no), cohabitation (yes/no), income (dichotomized at the median) and level of education (<10, 10–12, >12 years). To avoid unstable results, RR analysis was performed only when at least 5 exposed events were observed. To ensure anonymity, only cells containing at least 5 observations are displayed. Adjusted analyses for stillbirth included one covariate at a time owing to the low number of exposed stillbirths. We performed a number of sensitivity analyses: 1) extending the medication exposure window to 6 months before conception; 2) excluding women (from the unexposed group) who redeemed a prescription between 6 months and 30 days before conception, 3) including only women (in the exposed group) who redeemed at least two prescriptions of antipsychotic medications during the pregnancy period and 4) because an induced abortion could potentially have ended in a spontaneous abortion, if the pregnancy had not already been terminated, we made a sensitivity analysis using Cox regression including induced abortion and censoring of the pregnancies, when the pregnancy was terminated.

To assess for potential confounding effects of factors related to the underlying disorders requiring treatment with antipsychotic medications during pregnancy, we estimated the relative risk of SA and stillbirth after exposure to antipsychotic medications during pregnancy, compared with pregnant women who had used antipsychotic medications during the preceding year but not 30 days prior to the estimated conception date and during the pregnancy.

As a second step to separate potential effects of the medications from a potential effect of the underlying disorder, we assessed the risk of SA after exposure to antipsychotic medication in women with a diagnosis of severe mental disorder and in women without a diagnosis of severe mental disorder, respectively.

As a third step to adjust for unmeasured shared environmental risk factors and genetic predisposition, we performed a stratified Cox regression analysis with robust variance estimation and included women in the cohort who had at least two pregnancies with discordant exposure status (e.g. exposed in the first pregnancy, but not in the second) and discordant outcome. The stratified Cox regression analysis included a separate stratum for each woman; thus, each woman had her own baseline rate function.

We performed analyses of pregnancies with high (>50% of DDD) and low (≤ 50% of DDD) exposure to antipsychotic medications, respectively. We performed the main analysis of SA for each individual type of antipsychotic medication. The number of cases was too small to perform these analyses for the risk of stillbirth.

Statistical analyses were performed using Stata 12 (StataCorp, Texas, USA).

## Results

### Use of antipsychotic medications in pregnant women

Among 1,005,319 pregnancies, 3,164 were exposed to antipsychotic medications. Of these pregnancies, 1,164 ended as an induced abortion, which left 2,000 exposed pregnancies for analysis. These pregnancies resulted in 407 SA, 1,574 live births and 19 stillbirths. Of the 1,002,155 non-exposed pregnancies, 176,734 ended in an induced abortion, which left 825,421 unexposed pregnancies for analysis. These pregnancies resulted in 114,312 spontaneous abortions, 707,594 live births and 3,515 stillbirths. Women taking antipsychotic medications during pregnancy were less educated, more likely to be living alone and more likely to have a history of psychiatric disorder or substance abuse than women unexposed to antipsychotic medications during pregnancy (Table 1).

**Table 1:** Characteristics of study participants (numbers refer to pregnancies studied).

Characteristics		Exposed to antipsychotic drugs (APD) (N = 3,164) Number (per cent)	Unexposed to APD (N = 1,002,155) Number (per cent)
Maternal age at conception	<21 years	213 (6.7%)	56,736 (5.7%)
	21–25 years	527 (16.7%)	159,487 (15.9%)
	26–30 years	798 (25.2%)	340,548 (34.0%)
	31–35 years	881 (27.8%)	297,454 (29.7%)
	≥36 years	745 (23.5%)	147,883 (14.8%)
Cohabitation	Yes	1693 (53.5%)	767,510 (76.6%)
	No	1462 (46.2%)	219,346 (21.9%)
	Missing	9 (0.3%)	15,299 (1.5%)
Income (percentiles)	0–20 percentile	892 (28.2%)	198,906 (19.8%)
	>20–40 percentile	1098 (34.7%)	198,704 (19.8%)
	>40–60 percentile	657 (20.8%)	199,144 (19.9%)
	>60–80 percentile	325 (10.3%)	199,475 (19.9%)
	>80–100 percentile	191 (6.0%)	199,610 (19.9%)
	Missing	NA (0.0%)	6316 (0.6%)
Maternal education (years)	<10 years	829 (26.2%)	109,081 (10.9%)
	10–12 years	1325 (41.9%)	308,456 (30.8%)
	>12 years	906 (28.6%)	549,147 (54.8%)
	Missing	104 (3.3%)	35,471 (3.5%)
Maternal psychiatric history*	Yes	2369 (74.9%)	61,084 (6.1%)
	No	795 (25.1%)	941,071 (93.9%)
	Missing	0 (0.0%)	0 (0.0%)
Maternal history of substance abuse	Yes	507 (16.0%)	5097 (0.5%)
	No	2657 (84.0%)	997,058 (99.5%)
	Missing	0 (0.0%)	0 (0.0%)
Maternal history of severe mental disorder**	Yes	794 (25.1%)	1942 (0.2%)
	No	2370 (74.9%)	1,000,213 (99.8%)
	Missing	0 (0.0%)	0 (0.0%)
Co-medication	Any medication	3164 (100.0%)	627,775 (62.6%)
	Antiepileptic drugs	371 (11.7%)	4397 (0.4%)
	Antidepressant drugs	1539 (48.6%)	20,522 (2.0%)
	Insulin	31 (1.0%)	4628 (0.5%)
Pregnancy outcome	Induced abortion	1,154	173,680
	Induced abortion due to fetal disease	10	3,054
	Spontaneous abortion	407	114,312
	Live birth	1,574	707,594
	Stillbirth	19	3,515

*Mother diagnosed with a psychiatric disorder before end of pregnancy (ICD-8: 290–315 and ICD-10: all F codes)

**Mother diagnosed with a severe psychiatric disorder before end of pregnancy (ICD-8: 296.1–296.8, 298.1 and 295; ICD-10: F20 and F30–31)

The number of redeemed prescriptions for any type of antipsychotic medication during pregnancy increased during the study period from 299.9/100,000 pregnancies in 1997 to 490.8/100,000 pregnancies. An increase was also seen for most individual drugs (S1 Table).

### Risk of spontaneous abortion

Pregnancies exposed to any type of antipsychotic medication had an overall 34% increased risk of ending in an SA compared with unexposed pregnancies after adjusting for several confounding factors (aRR = 1.34; 95% confidence interval (CI) = 1.22; 1.46) (Table 2). An analysis restricting exposed women to include only those who redeemed at least two prescriptions during the pregnancy period showed a comparable estimate (aRR = 1.23; 95% CI = 1.08; 1.40).

**Table 2:** Relative risks of spontaneous abortion following APD exposure in pregnancy (numbers refer to pregnancy outcomes studied).

	APD exposure	Number	Spontaneous abortions, number (%)	Relative risk, crude (95% CI)	Relative risk, adjusted (95% CI)
Overall	Exposed	1881^$^	407 (21.6%)	1.59 (1.46; 1.74)	1.34* (1.22; 1.46)
	Unexposed	841,183	114,314 (13.6%)	1.00 (ref)	1.00 (ref)
Exposed to APD	Exposed during pregnancy	1881	407 (21.6%)	1.08 (0.96; 1.21)	1.04* (0.93; 1.17)
	Exposed only prior to pregnancy	2745	552 (20.1%)	1.00 (ref)	1.00 (ref)
Diagnosis of severe mental disorder	Exposed	461	109 (23.6%)	1.20 (0.98; 1.47)	1.14^#^ (0.94; 1.39)
	Unexposed	1337	263 (19.7%)	1.00 (ref)	1.00 (ref)
No diagnosis of severe mental disorder	Exposed	1420	298 (21.0%)	1.55 (1.39; 1.71)	1.34^#^ (1.21; 1.49)
	Unexposed	839,846	114,051 (13.6%)	1.00 (ref)	1.00 (ref)

^$^ Number lower than Table 1 due to shorter exposure time

* Adjusted for maternal age divided into three groups, history of drug abuse, cohabitation, income (above or below the median) and education (divided like in Table 1)

^#^Adjusted for maternal age divided into three groups, history of drug abuse, cohabitation and income (above or below the median)

Some women may have redeemed prescriptions before the pregnancy period, but taken the medication during pregnancy. To account for this, we performed additional analyses. In a sensitivity analysis extending the exposure period to 6 months before pregnancy, the risk estimate was similar (aRR = 1.33; 95% CI = 1.24; 1.42). The risk estimate was also similar in a sensitivity analysis excluding women from the unexposed group who redeemed a prescription during the 6 months before conception (aRR = 1.34; 95% CI = 1.22; 1.46). In a Cox regression analysis including induced abortions, the hazard ratio (HR) for spontaneous abortion was almost the same (adjusted HR = 1.25; 95% CI = 1.13; 1.39) as the RR in the main analysis.

In an attempt to separate the effect of the medication from the effect of the underlying disorder, we compared exposed pregnancies with pregnancies in which the woman was exposed to antipsychotic medications prior to pregnancy, but not during the pregnancy period. In this analysis, the relative risk of SA was close to one (aRR = 1.04; 95% CI = 0.93; 1.17) (Table 2).

In order to further disentangle the effects of medication from the effects of the underlying disorder, we performed a subanalysis of 323 women who had been pregnant at least twice during the study period, had used antipsychotics in at least one pregnancy and had been unexposed in at least one pregnancy and had experienced at least one SA (N = 994 pregnancies). We found the risk of SA in pregnancies exposed to antipsychotic medications rather similar to the risk in unexposed pregnancies in the same women (aHR = 1.11; 95% CI = 0.94; 1.31) (Table 3).

**Table 3:** Association between the use of APD and SA in pregnancies in 323 women having had at least two pregnancies and at least one abortion.

APD exposure	Number of pregnancies	Spontaneous abortion, number	Hazard ratio, crude (95% CI)	Hazard ratio, adjusted* (95% CI)
Exposed to APD	364	177	1.04 (0.88; 1.22)	1.11 (0.94; 1.31)
Unexposed to APD	630	282	1.00 (ref)	1.00 (ref)

* Adjusted for maternal age divided into three groups, history of drug abuse, cohabitation, income (above or below the median) and education (divided like in Table 1)

When the analyses were stratified according to a diagnosis of severe mental disorder, the risk of SA after APD exposure during pregnancy was increased, but not statistically significant (aRR = 1.14; 95% CI = 0.94; 1.39), in women with a diagnosis of severe mental disorder. However, in women without a diagnosis in the psychiatric registry (e.g. potentially treated exclusively by a GP), exposed pregnant women did have an increased risk (aRR = 1.34; 95% CI = 1.21; 1.49) (Table 2).

The vast majority of women who redeemed antipsychotic medications during pregnancy redeemed prescriptions for mean dose levels of less than 50% of DDD (92.7%). The aRR was increased three-fold in pregnancies exposed to a high dose (aRR = 3.19; 95% CI = 2.65; 3.84) and 36% in pregnancies exposed to a low dose of antipsychotic medications (aRR = 1.36; 95% CI = 1.22; 1.51) (Table 4). When we restricted these analyses to include only women with a diagnosis of severe mental disorder, we found a significantly increased risk only for use of high dose, but not for low (crude RR = 2.22; 95% CI = 1.67; 2.95 and crude RR = 0.96; 95% CI = 0.73; 1.26, respectively).

**Table 4:** Relative risk of SA following high and low dose of APD exposure in pregnancy (numbers refer to pregnancy outcomes).

APD dose	Number	Spontaneous abortion, number	Relative risk, crude (95% CI)	Relative risk, adjusted* (95% CI)
High	117	56	3.52 (2.91; 4.26)	3.19 (2.65; 3.84)
Low	1487	285	1.41 (1.27; 1.57)	1.36 (1.22; 1.51)
No exposure	841,183	114,314	1.00 (ref)	1.00 (ref)

*Adjusted for maternal age divided into three groups

The risk estimates for SA were rather similar for several types of antipsychotic medications (Table 5). However, CIs were wide and numbers were too small to perform adjusted analyses for most types of drugs.

**Table 5:** Relative risk of SA following exposure to individual APD (numbers refer to pregnancy outcomes).

APD	Exposure	Number	Spontaneous abortions, number	Relative risk, crude (95% CI)	Relative risk, adjusted* (95% CI)
Chlorprothixene	Total	365	99	2.00 (1.69; 2.36)	1.65 (1.39; 1.95)
	No exposure	841,183	114,314	1 (ref)	1 (ref)
Flupentixol	Total	233	56	1.77 (1.39; 2.24)	1.55 (1.22; 1.97)
	No exposure	841,183	114,314	1 (ref)	1 (ref)
Perphenazine	Total	229	44 (19.2%)	1.41 81.08; 1.86)	1.25 (0.95; 1.64)
	No exposure	841,183	114,314 (13.6%)	1 (ref)	1.(ref)
Zuclopenthixol	Total	229	43 (18.8%)	1.38 (1.05; 1.82)	1.26 (0.95; 1.66)
	No exposure	841,183	114,314 (13.6%)	1 (ref)	1 (ref)
Levomepromazine	Total	200	42 (21.0%)	1.55 (1.18; 2.03)	1.32 (1.01; 1.72)
	No exposure	841,183	114,314 (13.6%)	1 (ref)	1 (ref)
Quetiapine	Total	174	42 (24.1%)	1.78 (1.36; 2.32)	1.65 (1.28; 2.15)
	No exposure	841,183	114,314 (13.6%)	1 (ref)	1 (ref)
Olanzapine	Total	223	39 (17.5%)	1.29 (0.96; 1.72)	1.10 (0.83; 1.46)
	No exposure	841,183	114,314 (13.6%)	1 (ref)	1 (ref)
Lithium	Total	104	26 (25.0%)	1.84 (1.30; 2.60)	NA
	No exposure	841,183	114,314 (13.6%)	1 (ref)	NA
Risperidone	Total	116	25 (21.6%)	1.59 (1.12; 2.25)	NA
	No exposure	841,183	114,314 (13.6%)	1 (ref)	NA
Aripiprazole	Total	45	15 (33.3%)	2.45 (1.62; 3.71)	NA
	No exposure	841,183	114,314 (13.6%)	1 (ref)	NA
Ziprasidone	Total	41	11 (26.8%)	1.97 (1.20; 3.25)	NA
	No exposure	841,183	114,314 (13.6%)	1 (ref)	NA
Haloperidone	Total	40	10 (25.0%)	1.84 (1.04; 3.26)	NA
	No exposure	841,183	114,314 (13.6%)	1 (ref)	NA
Prochlorperazine	Total	74	7 (9.3%)	0.69 (0.34; 1.40)	NA
	No exposure	841,183	114,314 (13.6%)	1 (ref)	NA
Fluphenazine	Total	14	5 (35.7%)	2.63 (1.27; 5.45)	NA
	No exposure	841,183	114,314 (13.6%)	1 (ref)	NA
Chlorpromazine	Total	38	5 (13.2%)	0.97 (0.44; 2.15)	NA
	No exposure	841,183	114,314 (13.6%)	1 (ref)	NA

* Adjusted for maternal age divided into three groups and history of drug abuse.

### Risk of stillbirth

Pregnancies exposed to any type of investigated antipsychotic medication had a more than twofold increased risk of stillbirth (crude RR = 2.27; 95% CI = 1.45; 3.55) compared with unexposed pregnancies. Due to small sample size, adjustments were performed for one variable at a time. Adjusting for maternal age, cohabitation, income, history of severe mental disorder or history of drug misuse one at a time changed the estimates only slightly. The risk was almost unchanged when comparing with women who redeemed prescriptions for antipsychotic medications before but not during pregnancy (RR = 2.06; 95% CI = 1.01; 4.19) (Table 6).

**Table 6:** Relative risks of stillbirth following APD exposure in pregnancy (numbers refer to pregnancy outcomes studied).

	APD exposure	Number	Stillbirth, number (%)	Relative risk, crude (95% CI)
Overall	Exposed	1616	19 (1.2%)	2.27 (1.45; 3.55)
	Unexposed	726,727	3771 (0.5%)	1.00 (ref)
Exposed to APD	Exposed during pregnancy	1474	18 (1.2%)	2.06 (1.01; 4.19)
	Exposed only before pregnancy	2193	13 (0.6%)	1.00 (ref)

## Discussion

In this large nationwide cohort study, the risk of SA was not significantly increased in women taking antipsychotic medications during pregnancy compared with women taking antipsychotic medications before, but not during, pregnancy. The findings also indicate, that the risk of SA in women who were pregnant more than once during the study period was similar for women who were exposed and women who were unexposed to antipsychotic medication. Therefore, the overall 34% higher risk of SA in pregnancies exposed to antipsychotic medications compared with unexposed pregnancies may be due to factors related to the underlying disease rather than the treatment.

The results are in line with previous findings from smaller studies, which found no increased risk of SA after prenatal exposure to atypical antipsychotic medications.[14, 17, 18] Thus the increased risk of SA in women taking antipsychotic medications during pregnancy may be more related to their health problem and social conditions than to the medication. Confounding by indication may arise although it is unclear why a mental disorder in itself could cause an SA. Lifestyle factors and comorbidity may be more likely explanations. Based on the present study, an increased risk cannot be ruled out in women taking high doses of antipsychotic medications through long periods of pregnancy due to a dose response or threshold effect. However, confounding by indication may also explain these findings, because higher doses of antipsychotic medications represent not only a higher dose, but potentially also more severe disorder and more comorbid conditions.

We found that the risk of stillbirth increased twofold after exposure to antipsychotic medications. A large Danish cohort study has demonstrated an increased risk of stillbirth in pregnant women with previous psychiatric admission, regardless of diagnosis. [3] However, this former study did not include information about medication. Furthermore, in the present study, we were able to compare women who redeemed prescriptions for antipsychotic medications during pregnancy and women who redeemed similar prescriptions earlier in life. Our findings indicate that there may be an increased risk of stillbirth after exposure to antipsychotic medications. Due to small number of cases, we were unable to simultaneously adjust for multiple confounders. However, adjusting for relevant confounders one at a time, did not change the estimates considerably. We did not have the data to perform an analysis of subsequent pregnancies discordant to antipsychotic medication exposure in the same women.

### Strengths and weaknesses

This population-based cohort included all recorded pregnancies in Denmark between 1997 and 2008. The study had almost complete follow-up owing to the nationwide Danish registries, which makes selection of study participants and loss to follow-up unlikely explanations for our findings.

Classification of outcome is based on registry data that are mandatory for all inpatient treatments. Health care in Denmark is free and universally available. All stillbirths and most clinically recognized SAs are likely to be treated in a hospital setting. Thus misclassification of outcome is likely to be low. However, an SA occurring very early in a pregnancy will generally not be recorded in the hospital registry. These incidences may be interpreted as a late menstrual period or treated outside the hospital system. If SAs are more likely to be missed in women taking antipsychotic medications during pregnancy, or if exposure may interfere with very early survival of the pregnancy, this might lead to an underestimation of the risk associated with use of antipsychotic medication. However, we found no signs of differential timing of these terminations within the exposure groups.

Exposure data are based on complete registration of all redeemed prescriptions in Denmark. We assumed that women who redeemed a prescription of antipsychotic medications also took the medicine. A previous study on compliance with prescribed medication in Danish pregnant women showed 80% compliance to antidepressant medications. [29] Compliance to antipsychotic medication may differ from that of antidepressant medication, but a sensitivity analysis which included only women who redeemed at least two prescriptions during pregnancy and an analysis using extended exposure window, did not change the results markedly. The Danish National Prescription Register does not include information on medication administered during inpatient admissions. However, psychiatric disorder severe enough to prompt inpatient treatment would usually lead to continued treatment after discharge. The dosage may thus be underestimated in these cases but exposure is unlikely to have been missed completely.

The medication exposure window was defined as the period from 30 days before the estimated conception date to one day prior to spontaneous abortion/stillbirth/birth. The 30 days prior to conception were included in order to investigate delayed medication effects and to account for prescriptions in use at the time of conception. We included the day prior to termination of the pregnancy in the exposure window. We did this even though an effect of medication this close to termination of the pregnancy may be unlikely, because we wanted to be sure to investigate any possible immediate effects of medication

The numbers of women with prescriptions for specific types of antipsychotic drugs were too low to allow for in-depth estimates of the effects of the individual drugs.

We included lithium in the study, because we wanted to assess all drugs included in the ATC group: “Antipsychotic medication N05A” in spite of different chemical structure and indications. The relative risk of SA after exposure to lithium is quite similar to the risk of other drugs, which suggests that it is acceptable to include it in the general analysis.

The Danish Psychiatric Central Register contains information on treatment at psychiatric hospital-based units in Denmark. However, data on diagnoses made by general practitioners or private psychiatrists are not included in the register. Only diagnoses recorded in the register can be adjusted for.

Induced abortions occur more frequently in women who take antipsychotic medications during pregnancy than in women who do not take antipsychotic medications. Induced abortion may act as a competing event with regard to SA. This could result in underestimation of the risk of exposure to antipsychotic medications. However, a Cox regression model with induced abortion censored at the time of termination showed almost the same results as our binomial regression analysis.

We adjusted for several confounding factors in the analyses of SA. Nevertheless, residual and uncontrolled confounding may remain, as we lacked information on some important potential confounders, including smoking, alcohol intake, illegal drugs and poor diet. The results of the analysis restricted to women with severe mental disorder and the discordant pregnancies analysis indicate that such residual confounding is, in fact, present.

Stillbirth is a rare outcome. Despite the considerable size of the cohort, the number of fetal deaths was small. Thus, we were unable to adjust for multiple confounders at the same time or to perform sub-analysis of the risk of stillbirth in multiple pregnancies in the same women.

### Implications

The risk of SA in women taking antipsychotic medications during pregnancy was quite similar to the risk in women taking antipsychotic medications prior to conception but not during the pregnancy, and was almost similar to the risk in unexposed pregnancies in the same women. Thus the increased risk found in women taking antipsychotic medications during pregnancy compared with women not taking antipsychotic medication, may be caused by confounding. In women taking high doses of antipsychotic medications, results indicated a possible increased risk due to the medication itself.

The risk of stillbirth was twofold higher in pregnancies exposed to antipsychotic medication.

Treatment of pregnant women with antipsychotic medications requires careful consideration of indications and close clinical monitoring. In addition, as low doses as possible should be used. Potentially harmful fetotoxic effects, other than fetal death, should also be studied.

### Ethics statement

The use of registry data, including the combinations of information, was approved by the Danish Data Protection Agency. All data were anonymized before analysis. According to Danish law, approval by a research ethics committee was not needed for the project as the data included only non-personally identifiable information.

## Supporting Information

S1 Table Use of antipsychotic medications in the period from 1997 to 2008. (DOC)

## References

[1] Nilsson E, Lichtenstein P, Cnattingius S, Murray RM, Hultman CM. Women with schizophrenia: pregnancy outcome and infant death among their offspring. Schizophr Res. 2002;58(2–3):221–9. . 12409162 10.1016/s0920-9964(01)00370-x

[2] Howard LM, Goss C, Leese M, Thornicroft G. Medical outcome of pregnancy in women with psychotic disorders and their infants in the first year after birth. Br J Psychiatry. 2003;182:63–7. . 12509320 10.1192/bjp.182.1.63

[3] King-Hele S, Webb RT, Mortensen PB, Appleby L, Pickles A, Abel KM. Risk of stillbirth and neonatal death linked with maternal mental illness: a national cohort study. Arch Dis Child Fetal Neonatal Ed. 2009;94(2):F105–10. 10.1136/adc.2007.135459 . 19000999

[4] Gissler M, Artama M, Ritvanen A, Wahlbeck K. Use of psychotropic drugs before pregnancy and the risk for induced abortion: population-based register-data from Finland 1996–2006. BMC Public Health. 2010;10:383 10.1186/1471-2458-10-383 20591182 PMC2914778

[5] Alexander GC, Gallagher SA, Mascola A, Moloney RM, Stafford RS. Increasing off-label use of antipsychotic medications in the United States, 1995–2008. Pharmacoepidemiol Drug Saf. 2011;20(2):177–84. 10.1002/pds.2082 21254289 PMC3069498

[6] Stephenson CP, Karanges E, McGregor IS. Trends in the utilisation of psychotropic medications in Australia from 2000 to 2011. Aust N Z J Psychiatry. 2013;47(1):74–87. 10.1177/0004867412466595 . 23144164

[7] Vigod SN, Seeman MV, Ray JG, Anderson GM, Dennis CL, Grigoriadis S, et al Temporal trends in general and age-specific fertility rates among women with schizophrenia (1996–2009): a population-based study in Ontario, Canada . Schizophr Res. 2012;139(1–3):169–75. 10.1016/j.schres.2012.05.010 . 22658526

[8] Newport DJ, Calamaras MR, DeVane CL, Donovan J, Beach AJ, Winn S, et al Atypical antipsychotic administration during late pregnancy: placental passage and obstetrical outcomes. Am J Psychiatry. 2007;164(8):1214–20. 10.1176/appi.ajp.2007.06111886 . 17671284

[9] Gentile S. Antipsychotic therapy during early and late pregnancy. A systematic review. Schizophr Bull. 2010;36(3):518–44. 10.1093/schbul/sbn107 18787227 PMC2879689

[10] Hironaka M, Kotani T, Sumigama S, Tsuda H, Mano Y, Hayakawa H, et al Maternal mental disorders and pregnancy outcomes: a clinical study in a Japanese population. J Obstet Gynaecol Res. 2011;37(10):1283–9. 10.1111/j.1447-0756.2010.01512.x . 21535304

[11] Lin HC, Chen IJ, Chen YH, Lee HC, Wu FJ. Maternal schizophrenia and pregnancy outcome: does the use of antipsychotics make a difference? Schizophr Res. 2010;116(1):55–60. 10.1016/j.schres.2009.10.011 . 19896335

[12] Sadowski A, Todorow M, Yazdani Brojeni P, Koren G, Nulman I. Pregnancy outcomes following maternal exposure to second-generation antipsychotics given with other psychotropic drugs: a cohort study. BMJ Open. 2013;3(7). 10.1136/bmjopen-2013-003062 23852139 PMC3710985

[13] Reis M, Kallen B. Maternal use of antipsychotics in early pregnancy and delivery outcome. J Clin Psychopharmacol. 2008;28(3):279–88. 10.1097/JCP.0b013e318172b8d5 . 18480684

[14] Habermann F, Fritzsche J, Fuhlbruck F, Wacker E, Allignol A, Weber-Schoendorfer C, et al Atypical antipsychotic drugs and pregnancy outcome: a prospective, cohort study. J Clin Psychopharmacol. 2013;33(4):453–62. 10.1097/JCP.0b013e318295fe12 . 23764684

[15] Johnson KC, LaPrairie JL, Brennan PA, Stowe ZN, Newport DJ. Prenatal antipsychotic exposure and neuromotor performance during infancy. Arch Gen Psychiatry. 2012;69(8):787–94. 10.1001/archgenpsychiatry.2012.160 . 22474072 PMC4714592

[16] Peng M, Gao K, Ding Y, Ou J, Calabrese JR, Wu R, et al Effects of prenatal exposure to atypical antipsychotics on postnatal development and growth of infants: a case-controlled, prospective study. Psychopharmacology (Berl). 2013;228(4):577–84. 10.1007/s00213-013-3060-6 . 23559219

[17] McKenna K, Koren G, Tetelbaum M, Wilton L, Shakir S, Diav-Citrin O, et al Pregnancy outcome of women using atypical antipsychotic drugs: a prospective comparative study. J Clin Psychiatry. 2005;66(4):444–9; quiz 546. . 15816786 10.4088/jcp.v66n0406

[18] Diav-Citrin O, Shechtman S, Ornoy S, Arnon J, Schaefer C, Garbis H, et al Safety of haloperidol and penfluridol in pregnancy: a multicenter, prospective, controlled study. J Clin Psychiatry. 2005;66(3):317–22. . 15766297 10.4088/jcp.v66n0307

[19] Twaites BR, Wilton LV, Shakir SA. The safety of quetiapine: results of a post-marketing surveillance study on 1728 patients in England. J Psychopharmacol. 2007;21(4):392–9. 10.1177/0269881107073257 . 17656426

[20] Goldstein DJ, Corbin LA, Fung MC. Olanzapine-exposed pregnancies and lactation: early experience. J Clin Psychopharmacol. 2000;20(4):399–403. . 10917399 10.1097/00004714-200008000-00002

[21] Biswasl PN, Wilton LV, Pearcel GL, Freemantle S, Shakir SA. The pharmacovigilance of olanzapine: results of a post-marketing surveillance study on 8858 patients in England. J Psychopharmacol. 2001;15(4):265–71. . 11769820 10.1177/026988110101500405

[22] Coppola D, Russo LJ, Kwarta RF Jr., Varughese R, Schmider J. Evaluating the postmarketing experience of risperidone use during pregnancy: pregnancy and neonatal outcomes. Drug Saf. 2007;30(3):247–64. . 17343431 10.2165/00002018-200730030-00006

[23] Bennedsen BE, Mortensen PB, Olesen AV, Henriksen TB. Congenital malformations, stillbirths, and infant deaths among children of women with schizophrenia. Arch Gen Psychiatry. 2001;58(7):674–9. . 11448375 10.1001/archpsyc.58.7.674

[24] Kildemoes HW, Sorensen HT, Hallas J. The Danish National Prescription Registry. Scand J Public Health. 2011;39(7 Suppl):38–41. 10.1177/1403494810394717 . 21775349

[25] Andersen TF, Madsen M, Jorgensen J, Mellemkjoer L, Olsen JH. The Danish National Hospital Register. A valuable source of data for modern health sciences. Dan Med Bull. 1999;46(3):263–8. . 10421985

[26] The ICD-10 classification of mental and behaviorural disorders: diagnostic criteria for research 1 ed. ed. World Health Organization: Geneva; 1993.

[27] Knudsen LB, Olsen J. The Danish Medical Birth Registry. Dan Med Bull. 1998;45(3):320–3. . 9675544

[28] Munk-Jorgensen P, Mortensen PB. The Danish Psychiatric Central Register. Dan Med Bull. 1997;44(1):82–4. . 9062767

[29] Olesen C, Sondergaard C, Thrane N, Nielsen GL, de Jong-van den Berg L, Olsen J, et al Do pregnant women report use of dispensed medications? Epidemiology. 2001;12(5):497–501. . 11505166 10.1097/00001648-200109000-00006

