# Supplementary material for: Risk of Fetal Death after Treatment with Antipsychotic Medications during Pregnancy
Source: PLoS One. 2015 Jul 10;10(7):e0132280. doi: 10.1371/journal.pone.0132280 (PMC4498617; doi:10.1371/journal.pone.0132280)
Supplement: S1 Table — (DOC) [file pone.0132280.s001.doc]

Table 1, supporting information

| ***Women in cohort receiving any antipsychotic treatment, pr. 100.000*** | ***1997*** | ***1998*** | ***1999*** | ***2000*** | ***2001*** | ***2002*** | ***2003*** | ***2004*** | ***2005*** | ***2006*** | ***2007*** | ***2008*** |
| --- | --- | --- | --- | --- | --- | --- | --- | --- | --- | --- | --- | --- |
| Any antipsychotic agent | 299.9 | 273.0 | 224.1 | 241.8 | 219.2 | 287.8 | 285.0 | 298.0 | 340.3 | 360.4 | 456.6 | 490.8 |
| Chlorprothixene | 56.4 | 45.9 | 32.0 | 35.0 | 37.3 | 51.8 | 57.7 | 76.5 | 83.9 | 98.9 | 128.8 | 134.7 |
| Flupentixol | 42.3 | 50.2 | 36.4 | 41.6 | 36.2 | 46.0 | 38.5 | 24.7 | 42.0 | 29.6 | 38.0 | 28.5 |
| Perphenazine | 42.3 | 33.8 | 24.3 | 21.9 | 20.3 | 26.5 | 35.1 | 19.1 | 26.1 | 29.6 | 48.3 | 77.6 |
| Zuclopenthixol | 74.1 | 40.4 | 32.0 | 37.2 | 32.8 | 44.9 | 33.9 | 41.6 | 34.0 | 25.0 | 26.5 | 25.1 |
| Levomepromazine | 35.3 | 45.9 | 37.5 | 32.8 | 28.2 | 33.4 | 29.4 | 32.6 | 39.7 | 25.0 | 32.2 | 24.0 |
| Quetiapine | 0 | 0 | 0 | 0 | NA | 11.5 | 20.4 | 13.5 | 34.0 | 68.2 | 90.9 | 142.7 |
| Olanzapine | NA | 5.5 | 14.4 | 21.9 | 26.0 | 31.1 | 32.8 | 42.7 | 53.3 | 53.4 | 58.7 | 52.5 |
| Lithium | NA | 16.4 | 22.1 | 13.1 | 18.1 | 17.3 | 18.1 | 6.7 | 21.6 | 18.2 | 24.2 | 25.1 |
| Risperidon | 0 | NA | 5.5 | 10.9 | 11.3 | 17.3 | 24.9 | 29.2 | 32.9 | 36.3 | 42.6 | 30.8 |
| Aripiprazole | 0 | 0 | 0 | 0 | 0 | 0 | 0 | NA | 18.1 | 33.0 | 36.8 | 30.8 |
| Ziprasidone | 0 | 0 | 0 | 0 | 0 | NA | 13.6 | 22.5 | 14.7 | 14.8 | 18.4 | 11.4 |
| Haloperidol | NA | NA | NA | 7.7 | 6.8 | 6.9 | 6.8 | 5.6 | NA | 6.8 | NA | NA |
| Prochlorperazin | 21.2 | 15.3 | 9.94 | 12.0 | 12.4 | 19.6 | 5.7 | 7.9 | NA | NA | 6.9 | NA |
| Fluphenazin | NA | NA | NA | NA | NA | 0 | NA | NA | 0 | NA | 0 | 0 |
| Chlorpromazin | NA | 15.3 | 7.7 | 9.8 | NA | 8.1 | NA | NA | 0 | 0 | 0 | 0 |
